# Physical growth and sexual maturation of perinatally HIV-infected adolescent males in a southeast Nigerian tertiary hospital: a comparative cross-sectional study

**DOI:** 10.1186/s12887-022-03626-2

**Published:** 2022-10-05

**Authors:** Chibuzo O Ndiokwelu, Samuel N Uwaezuoke, Kenechukwu K Iloh

**Affiliations:** 1 Department of Pediatrics, University of Nigeria Teaching Hospital (UNTH), Ituku-Ozalla Enugu, Enugu, Nigeria; 2 College of Medicine, University of Nigeria Enugu Campus, Enugu, Nigeria

**Keywords:** Adolescent males, Acquired Immune Deficiency Syndrome, Anthropometry, Sexual maturation, Nigeria

## Abstract

**Background:**

The advent of highly-active anti-retroviral therapy (HAART) has resulted in the survival of children with Human Immunodeficiency Virus (HIV)/Acquired Immune Deficiency Syndrome (AIDS) into adolescence. Their prolonged survival has translated into co-morbidities like endocrine deficiencies which may manifest as growth and pubertal delay. This study aimed to determine the physical growth and sexual maturation of perinatally HIV-infected adolescent males and compare them with those of age-matched HIV-negative controls.

**Methods:**

We conducted a comparative cross-sectional study of 104 perinatally HIV-infected males on HAART aged 10 to 19 years, and 104 age-matched HIV-negative males who served as controls. The subjects and controls were enrolled and assessed at a Nigerian tertiary hospital over six months. Anthropometric measurements such as weight, height, and BMI were obtained and Z scores for age were derived for weight, height, and BMI to determine physical growth using WHO AnthroPlus software. Sexual maturation was assessed using the method proposed by Marshall and Tanner. Data analysis and appropriate statistics were conducted with the Statistical Package for Social Sciences (SPSS) version 25 Chicago IL. A *p*-value < 0.05 was adopted as the level of statistical significance.

**Results:**

The mean height, weight, and BMI Z scores of the subjects were all lower than those of the controls. The difference between the mean weight of the subjects (44.60 ± 13.32 kg) and the controls (49.97 ± 13.58 kg) was statistically significant (t = 2.88, *p* = 0.004). Similarly, the difference between the mean BMI Z-scores of the subjects (-0.96 ± 1.95) and the controls (-0.10 ± 0.86) was statistically significant (t = 4.10, *p* = < 0.001). The subjects showed a delay in pubic hair and testicular development for Stages 1, 2, and 3. Duration of HAART did not significantly affect the BMI of subjects who were in three groups: undernutrition, normal nutrition, and overnutrition (Kruskal-Wallis test, *p* = 0.30).

**Conclusion:**

Perinatal HIV infection negatively affects physical growth and the onset of pubic-hair development (PH 2) despite the duration of HAART. We recommend that screening for weight deficit or pubertal delay should form part of the management protocol for HIV-infected male children on HAART.

## Background

As a global pandemic, the Human Immunodeficiency Virus (HIV) infection has become a major contributor to childhood morbidity and mortality in sub-Saharan Africa. Mother-to-child transmission accounts for about 90% of pediatric HIV infections in sub-Saharan Africa [1]. The majority of perinatally HIV-infected children in Africa present with HIV-related symptoms by six months of age; the disease progresses rapidly with up to 50% of infected children developing Acquired Immune Deficiency Syndrome (AIDS) and experiencing mortality within the first two years of life in the absence of intervention [2]. However, the advent of the prevention of mother-to-child transmission (PMTCT) of HIV has resulted in a significant number of perinatally HIV-infected children surviving into adolescence [3].

Symptomatic HIV infection is associated with an early and progressive reduction in weight and pubertal growth spurt, which can affect sexual maturation and result in delayed puberty [4, 5]. Delayed growth and puberty are particularly common in chronic diseases such as HIV/AIDS, and more frequently in adolescents with malnutrition and chronic inflammation [6]. HIV infection can contribute to both linear growth and weight-gain disturbances in early childhood which appear as early as three months of age [7]. The growth deficits continue to be observed as these HIV-infected children grow into adolescence. Although the exact mechanisms for the growth retardation remain speculative, some authors suggest that proteolysis of insulin-like growth factor-1 binding protein-3 (IGFBP-3) may diminish the function of insulin-like growth factor-1 (IGF-1): resulting in growth retardation [8]. Other authors had previously reported that IGF-1 levels were reduced in HIV-infected children [9, 10]. A collaborative comparative study in Europe conducted in eleven centers across eight European countries specifically documented an average weight and height deficits of 7 kg and 7.5 cm, respectively by 10 years of age among HIV-infected children compared to their HIV-uninfected counterparts [11].

Similarly, the mechanisms of pubertal delay in HIV-infected children are still not fully understood. However, several factors generally referred to as dysfunction of the hypothalamic-pituitary-gonadal (HPG) axis (which includes growth-hormone dysregulation, hypothyroidism, and testosterone reduction) have been implicated in the pathophysiology [12]. These endocrine deficiencies occur as a result of direct HIV invasion, lymphocytic infiltration, and opportunistic infections. The euthyroid sick syndrome (accompanied by increased basal thyrotropin levels, free thyroxin levels, low IGF-1, and IGF BP-3 due to HIV-triggered production of pro-inflammatory cytokines) has been identified as a possible mechanism leading to delayed sexual maturation [13].

Whereas studies demonstrating growth and pubertal delay in male and female HIV-infected children have been conducted in the developed settings of Europe and the United States [14–17], similar studies in either of the sexes or both sexes have been conducted in sub-Saharan Africa as well [18–22]. In southeast Nigeria, a previous study in this locality focused on the female sex alone by investigating sexual maturation in perinatally HIV-infected girls aged 8–17 years [23]. The present study, however, aimed to determine the physical growth and sexual maturation of perinatally HIV-infected adolescent males and compare them with those of age-matched HIV-negative controls.

## Methods

### Study design and site

This study was designed as a hospital-based, comparative cross-sectional study conducted at the University of Nigeria Teaching Hospital (UNTH), Ituku-Ozalla in Enugu over six months (September 2019 - February 2020). It was conducted in the Pediatric HIV and Out-Patient Clinics of the hospital. A total of 262 children aged 10 to 19 years were registered in this Pediatric HIV Clinic, comprising 117 males (112 perinatally infected) and 145 females as of September 2019. The clinics attended to children up to 19 years because there was also a specialty Paediatric Adolescent Clinic which worked synergistically with other clinics during this transition phase in guiding the patients’ transition to the equivalent adult clinics.

### Study population and sampling method

The study subjects were adolescent males attending HIV and Children Out-Patient Clinics. Perinatally HIV-infected males (aged 10–19 years) on highly-active anti-retroviral therapy (HAART) were included as the study subjects. The controls were healthy HIV-negative males (aged 10–19 years) who had no acute illness for at least 2 weeks before recruitment. They were patients on follow-up visits after recovery from curable infectious diseases (such as malaria and acute respiratory infections). They had pre-test and post-test HIV counseling sessions which were conducted routinely by a trained dedicated counselor from the HIV Clinic working synergistically with the Children Out-patient Clinic for on-site counseling and testing. Additionally, these controls were investigated for HIV in two sequential screening tests. Participants excluded from the study consisted of the following: HIV-infected males (aged 10–19 years) on long-term medications that could affect growth and sexual maturation such as steroids and anti-epileptic drugs; HIV-infected males (aged 10–19 years) with chronic diseases like bronchial asthma, diabetes mellitus, sickle cell anemia, cancer, chronic kidney disease, or other chronic cardiac, respiratory, renal, and endocrine disorders; HIV-negative males (aged 10–19 years) with similar chronic diseases; and HIV-negative males (aged 10–19 years) on similar long-term medications that could affect growth and sexual maturation. After informed consent from parents/caregivers and participants’ assent, the perinatally HIV-infected males and their sex-and age-matched HIV-negative controls were recruited by consecutive sampling in the Pediatric HIV and the Pediatric Out-Patient Clinics, respectively until the desired sample size was achieved.

### Study procedure

A case-record form that required information about the subjects’ socio-demographic profile, and other relevant data extracted from their hospital records, was completed during the study. The social classification of the study participants was based on the proposed method that utilizes parental education and occupation [24]. The social classes were re-grouped as the upper class (classes 1 and 2), middle class (class 3), and lower class (classes 4 and 5). The participants’ physical growth characteristics were assessed with height and weight measurements by one of the authors (CON) according to standardized procedures. BMI was calculated using the formula: weight (kg)/height (m^2^). The weight-for-age, height-for-age, and Z scores for weight, height, and BMI derived from WHO reference values and WHO AnthroPlus software was obtained. The sexual maturity rating of the participants was conducted manually by CON using a Prader orchidometer to measure the testicular volume and standardized photographs to grade the pubic hair pattern. The participants’ pubertal stages were also assessed by CON, based on the proposed method by Marshall and Tanner [25]. Using pubic hair, the staging comprised Stage 1 (PH1): no hair, Stage 2 (PH2): scanty, long, and slightly pigmented hair, Stage 3 (PH3): darker, starting to curl, small amounts of hair, Stage 4 (PH4): resembling adult-type but less quantity, coarse, and Stage 5 (PH5): adult distribution and spread to the medial surfaces of the thighs. For testicular volume (average volume), the staging was made up of Stage 1 (G1):3 ml. (< 4 ml.), Stage 2 (G2): 4–6 ml. (4 ml.), Stage 3 (G3): 6–12 ml. (10 ml.), Stage 4 (G4): 12–20 ml. (16 ml.), and Stage 5 (G5) : >20 ml. (25 ml.).

### Ethical considerations

Study participants were only enrolled after consent and assent were obtained from the parents/guardians and the participants, respectively. Confidentiality was emphasized and maintained throughout the study. Participants were anonymized and identified with coded initials on the case-record forms.

### Data analysis

We conducted data analysis using the Statistical Package for Social Sciences (SPSS) version 25 (Chicago Illinois) for Windows. Data were scrutinized for incorrectly filled information and cleaned. The normality of data distribution was checked using the Shapiro-Wilk test. The results were displayed in frequencies, charts, and tables as appropriate. Normally distributed data [e.g. height, weight, height-for-age Z-score (HAZ), body mass index-for-age Z-score (BAZ)] were analyzed by t-test. Non-normally distributed data (duration of HAART use in three groups of HIV-positive subjects categorized with their BMI as undernutrition, normal nutrition, and overnutrition) was analyzed by using the Kruskal-Wallis test. Comparisons of the anthropometric variables (e.g. height, weight, HAZ, and BAZ) between the HIV-infected subjects and the controls were also analyzed using an independent t-test. All tests of significance were two-tailed at a 5% level of significance (p = 0.05) and 95% confidence interval (CI).

## Results

### Socio-demographic profile of study participants

According to age distribution (as shown in Table 1), 51 (49.0%) of the 104 subjects and 51 (49.0%) of the 104 controls were aged 10–14 years (early adolescence). The remaining 53 (51.0%) of the subjects and 53 (51.0%) of the controls were aged 15–19 years (late adolescence). The estimated mean ages for both subjects and controls were similar: 14.38 **±** 2.68 years. Regarding socio-economic status (SES), 67.3% of the subjects that were HIV-positive belonged to the lower SES (classes 4 and 5) compared to 15.4% of their HIV-negative counterparts. The difference in SES between the two groups was statistically significant (p < 0.001). Specifically, the HIV-positive subjects were about 19 times more likely to belong to the lower SES than the HIV-negative controls [OR (95% CI) = 19.5(8.7–43.9)].

Furthermore, an assessment of the living status of subjects’ parents (mothers and fathers) shows that the majority (62.0%) of the perinatally HIV-infected subjects were orphans compared to their HIV-negative controls (23%). This disparity may be attributed to parents’ HIV-related mortality given that the subjects’ HIV infections were vertically transmitted.

### Clinical and laboratory profile of the HIV-infected subjects

The diagnosis of HIV infection in all the infected adolescents was made before they attained the age of 5 years. The median duration of HAART use as at the time of the study was between 8 and 9.5 years. The treatment regimen which all the subjects were taking during the period of study was the one based on WHO-guideline for children < 10 years and adolescents: abacavir or tenofovir and lamivudine (two nucleoside analog reverse-transcriptase inhibitors) plus efavirenz (a non-nucleoside reverse-transcriptase inhibitors). The CD4 + count (estimated within the six months of subjects’ enrolment) was used for immunological staging of infection severity: Stage 1 representing CD4 + count of > 500 cells/mm^3^, Stage 2, CD4 + count of 350–499 cells/mm^3^, Stage 3, CD4 + count of 200–349 cells/mm^3^ and Stage 4, CD4 + count of < 200 cells/mm^3^. The subjects were distributed as follows: 79 adolescents (76.0%) in Stage 1, 16 (15.4%) in Stage 2, 4 (3.8%) in Stage 3 and 5 (4.8%) in Stage 4.

**Table 1:** Comparison of socio-demographic profile of HIV-infected subjects and HIV-negative controls

Variables	Subjects n (%)	Controls n (%)	Test statistic	p-value	OR (95% CI)
Socio-economic status (SES) -Upper SES (Classes 1 & 2) -Middle SES (Class 3) -Lower SES (Classes 4 & 5)	13 (12.5) 21 (20.2) 70 (67.3)	58 (55.8) 30 (28.8) 16 (15.4)	FT	< 0.001*	**--** 3.1 (1.4–7.1) 19.5 (8.7–43.9)
Age distribution^†^ -Early adolescence (10–14 years) -Late adolescence (15–19 years)	51 (49.0) 53 (51.0)	51 (49.0) 53 (51.0)			-- --
Living with parents -No (Orphans) -Yes (Non-orphans)	64 (62.0) 40 (38.0)	24 (23.0) 80 (77.0)			-- --

**FT**, Fisher’s exact test **HIV**, Human Immunodeficiency Virus **OR**, Odds ratio **CI**, Confidence interval

*Statistically significant ^**†**^Mean age of subjects and controls = 14.38 **±** 2.68 years

### Anthropometric parameters of the study participants

As illustrated in Table 2, the difference between the mean height of the HIV-infected subjects (154.93 ± 14.78 cm, range 125.90-179.60 cm) and that of the HIV-negative controls (157.16 ± 10.77 cm, range 131.80-177.50 cm) was not significant (p = 0.22). However, the difference between the weight of the subjects (44.60 ± 13.32 kg, range: 22.30–71.30 kg) and that of the controls (49.97 ± 13.58 kg, range: 27.10–70.80 kg) was statistically significant (p = 0.004). The difference between the mean height Z score of the subjects and that of the controls was not statistically significant (p = 0.13) while the difference between the mean BMI and BMI-for-age Z (BAZ) Scores of the subjects (18.58 ± 3.78 vs. -0.96 ± 1.95) and the controls (20.82 ± 4.03 vs. -0.10 ± 0.86) was statistically significant (p < 0.001). Also, the calculated effect sizes of the variables indicate a strong effect (effect size > 0.5) from mean BMI and BAZ, implying that the impact of HIV infection was most obvious on these anthropometric indices.

**Table 2:** Comparison of the anthropometric variables between the HIV-infected subjects and HIV-negative controls

Variables	Subjects (n = 104)	Controls (n = 104)	Test statistic	p-value	Mean difference	95% CI of mean difference
Mean height (cm)^†^	154.93 ± 14.78	157.16 ± 10.77	t= −1.24	0.22	−2.23	−5.77-1.30
Mean weight (kg)^‡^	44.60 ± 13.32	49.97 ± 13.58	t= −2.88	0.004*	−5.36	−9.04- 1.69
Mean BMI^∞^	18.58 ± 3.78	20.82 ± 4.03	t= −4.15	< 0.001 *	−2.24	−0.39-0.49
HAZ ^ξ^	−1.16 ± 0.92	−0.98 ± 0.83	t= −1.54	0.13	−0.19	−0.43-0.05
BAZ ^§^	−0.96 ± 1.95	−0.10 ± 0.86	t= -4.10	< 0.001*	−1.85	−2.56-1.14

**HIV**, Human immune-deficiency virus **CI**, Confidence interval **BMI**, body mass index **HAZ**, Height-for-age Z score **BAZ**, Body mass index-for-age Z score **t**, independent t-test *Statistically significant ^†^Effect size = 0.21 ^‡^Effect size = 0.40 ^ξ^ Effect size = 0.22 ^§^Effect size = 1^∞^Effect size = 0.56

### Sexual maturation of the study participants

The sexual maturation of the HIV-positive subjects and their HIV-negative controls is shown in Table 3. The difference in the sexual maturation between the two groups was significant (pubic hair, p = 0.001 and testicular volume, p = 0.008). Notably, the subjects were ten times less likely to achieve pubic hair development (stage 2) than the controls [OR (95% CI) = 0.1(0.1–0.5)]. They, however, were about three times more likely to achieve full testicular development (stage 5) than the controls [OR (95% CI) = 2.7(1.2–6.1)].

**Table 3:** Comparison of the sexual maturation between HIV-positive subjects and HIV-negative controls

Variables	Subjects (n = 104)	Controls (n = 104)	Test Statistic p-value	OR (95% CI)
Public hair Stage 1 Stage 2 Stage 3 Stage 4 Stage 5	14(13.5%) 25(24.0%) 18(17.3%) 22(21.2%) 25(24.0%)	0(0.0%) 34(32.7%) 24(23.1%) 30(28.8%) 16(15.4%)	FT 0.001*	- 0.1(0.1–0.5) 1.1(0.5–2.3) 1.0(0.4–2.2) 2.1(0.9–4.9)
Testicular volume Stage 1 Stage 2 Stage 3 Stage 4 Stage 5	2(1.9%) 31(29.8%) 24(23.1%) 18(17.3%) 29(27.9%)	0(0.0%) 18(17.3%) 32(30.8%) 34(32.7%) 20(19.2%)	FT 0.008*	- 0.3(0.1–7.5) 0.4(0.2–1.0) 0.7(0.3–1.5) 2.7(1.2–6.1)

**FT**, Fisher’s exact test **CI**, Confidence interval

*Statistically significant

### SES and duration of HAART versus physical growth

The association of the subjects’ SES and duration of HAART with their physical growth was evaluated (Table 4). Interestingly, there was no significant relationship between the subjects’ SES and physical growth on one hand (p = 0.57), and duration of HAART and physical growth on the other hand (p = 0.30).

**Table 4:** Relationship between the subjects’ socioeconomic status (SES)/duration of HAART and physical growth using body mass index

Variables	Undernutrition^†^	Normal nutrition	Overnutrition^‡^	N = 104	Test statistic	p-value
SES Upper (Classes 1 and 2) Middle (Class 3) Lower (Classes 4 and 5)	5(38.5%) 5(23.8%) 21(30.0%)	8(61.5%) 14(66.7%) 39(55.7%)	0(0.0%) 2(9.5%) 10(14.3%)	13(100.0) 21(100.0) 70(100.0)	FT	0.57
Duration of HAART use Median years (IQR)	8.00(4.00)	9.00(6.00)	9.50(2.00)		1.34	0.30*

**NB**: BMI-for Age Z score (BAZ) was used to assess nutritional status (Severe thinness = BAZ < -3; Thinness = BAZ − 3 to < -2; Normal = BAZ − 2 to + 1 Overweight = BAZ > + 1 to + 2; Obesity = BAZ > + 2)

^†^Undernutrition = Thinness + Severe thinness

^‡^Overnutrition = Overweight + Obesity

IQR, Interquartile range, * Kruskal Wallis test, FT, Fisher’s exact test.

### SES and duration of HAART versus sexual maturation

The association of the subjects’ SES and duration of HAART with sexual maturation was assessed, as shown in Table 5. The majority of HIV-infected children in the different stages of pubic hair development and testicular volume belonged to the lower SES. The relationship between their SES and sexual maturation was significant (p < 0.001). Similarly, there was a significant relationship between the duration of HAART and sexual maturation using testicular volume as the index of sexual maturity (p = 0.02). A Post-hoc pairwise comparison showed that the difference was between stage 1 and stage 5.

**Table 5:** Relationship between the socio-economic status (SES)/duration of HAART and sexual maturation N = 104

Variables	Sexual maturation using pubic hair					Test Stat. p-value
	Stage 1	Stage 2	Stage 3	Stage 4	Stage 5	
SES Upper Middle Lower Total	0(0.0%) 2(14.3%) 12(85.7%) 14(100.0)	2(8.0%) 8(32.0%) 15(60.0%) 25(100.0%)	0(0.0%) 1(5.6%) 17(94.4%) 18(100.0%)	1(4.5%) 5(22.7%) 16(72.7%) 22(100.0%)	10(40.0%) 5(20.0%) 10(40.0%) 25(100.0%)	FT <0.001
Duration of HAART use Median years (IQR)	7.00(3.50)	9.00(3.00)	9.00(11.00)	9.00(8.00)	10.00(8.00)	χ^2^ = 1.21 0.19*
	Sexual maturation using testicular volume					
SES Upper Middle Lower Total	0(0.0%) 2(100.0%) 0(0.0%) 2(100.0)	0(0.0%) 4(12.9%) 27(87.1%) 31(100.0%)	2(8.3%) 5(20.8%) 17(70.8%) 24(100.0%)	1(5.6%) 3(16.7%) 14(77.8%) 18(100.0%)	10(34.5%) 7(24.1%) 12(41.4%) 29(100.0%)	FT <0.001
Duration of HAART use Median years (IQR)	6.00(0.00)	8.00(9.00)	9.50(6.25)	7.00(6.50)	11.00(5.50)	χ^2^ = 3.12 0.02*

*Kruskal Wallis test, IQR, Interquartile range FT, Fisher’s exact test χ^2^, Chi-square test

### The effect of BMI, duration of HAART use, and SES on testicular volume after controlling for age

The effect of the independent variables (e.g., BMI, and SES) on testicular volume after controlling for age is shown in Table 6. Specifically, the effect of nutritional status (measured with BMZ), duration of HAART use, and SES on testicular volume was weak with effect-size values of 0.007, 0.006, and 0.127, respectively. Thus, their effect was adjudged insignificant. However, age as a covariate had a strong effect (> 0.5) on testicular volume (effect size = 0.694).

**Table 6:** Analysis of covariance (ANCOVA) showing the effect of nutritional status (using BMI) and SES on testicular volume after adjusting for age Dependent Variable: Stage of testicular volume

Source	Type III Sum of Squares	Df	Mean Square	F	Sig.	Partial Eta-squared
Corrected Model	120.277^a^	9	13.364	34.361	0.000	0.767
Intercept	10.232	1	10.232	26.309	0.000	0.219
Age	84.998	1	82.989	213.401	0.000	0.694 *
Duration of HAART	0.229	1	0.229	0.588	0.445	0.006 *
BMZ	0.254	2	0.127	0.326	0.722	0.007 *
SES	5.316	2	2.685	6.835	0.002	0.127 *
BMZ x SES	2.537	3	0.846	2.175	0.096	0.065
Error	36.559	94	0.389			
Total	1355.000	104				
Corrected Total	156.837	103				

R Squared = 0.767 (Adjusted R Squared = 0.745), BMZ = BMI Z-score. * Effect size values of age (as covariate) and independent variables: ≤0.2 = small/weak effect, ≥ 0.5 = medium/large effect.

Sig. =significance.

## Discussion

The physical growth and sexual maturation of perinatally-HIV infected male and female children have been well documented in studies conducted in developed and developing countries. In Nigeria, the few published studies that evaluated these outcomes were conducted in perinatally HIV-infected girls alone [20, 23]. Thus, it is important to obtain data on similar outcomes among adolescent males seen in this clime.

In the present study, we assessed the physical growth and sexual maturity rating of perinatally HIV-infected adolescent males using anthropometric indices and grading of pubic-hair/testicular volume, respectively; and compared these parameters with those of age-matched HIV-negative controls. Firstly, we found that HIV-infected subjects had significantly lower weight and BMI Z-scores compared to their HIV-negative controls despite the use of HAART. Previous studies have reported varying degrees of growth deficits in height and weight among HIV-infected children [21, 26–28]. For instance, some authors in South Africa documented suboptimal growth in adolescents with perinatally-acquired HIV infection: which appeared more pronounced in males than in females, in those who commenced HAART later in life, and in those who had baseline stunting [21]. Other authors in the same country reported that perinatally HIV-infected children had early and sustained stunting, characterized by persistent low mean Z-scores for length-for-age and weight-for-age whereas those with rapidly progressive disease had both stunting and wasting [26]. Additionally, a cohort study in the West African subregion noted that growth evolution was particularly strong among HIV-infected children on HAART within the first 2 years but declined after this period; the investigators specifically reported initial gains in WAZ and HAZ within 12 and 24 months, respectively which slowed down subsequently [27]. Similarly, another cohort study in Malawi (Southern Africa) observed that although the growth trajectories of HIV-infected children on HAART showed sustained positive response among those continuing on therapy, normal WAZ and HAZ values were not eventually attained: implying that weight loss and stunting were the ultimate outcomes in these children [28]. The findings of these studies [21, 26–28] are consistent with those of the present study although we found no significant difference in HAZ values of HIV-infected and HIV-negative children: an observation that is in tandem with the reports of other authors [29, 30]. We believe that the absence of a significant difference in HAZ values in our study cohorts may be explained by the prevalence of a high level of stunting in the general population in Nigeria [31]. This high level of stunting means that differences in height between HIV-infected children and the general population may not, therefore, be significant. Based on our findings that showed a significant negative effect of HIV infection on weight and BMI Z-score, we suggest that these weight deficits can be attributed to HIV-induced immune dysfunction, attendant intercurrent infections, preferential decreases in lean body mass [32], and growth hormone resistance [33, 34]. Our finding also underscores the fact that regardless of racial and population differences or geographical location, HIV infection adversely affects weight gain. Although the exact primary mechanisms by which HIV impedes growth have not been fully established, secondary causes of growth faltering, such as dietary insufficiency and diarrhoeal illnesses, may be involved [35]. Even without secondary infection, whole-body protein kinetics are altered with increased protein catabolism and synthesis of acute-phase reactants in response to viral replication, thus diverting energy away from the accrual of fat-free mass and growth [35]. HIV-infected children have also been noted to accumulate bone density more slowly than non-infected children through direct infection of bone cells causing elevation of several cytokines (interleukin 1, interleukin 6, and tumor necrosis factor-α) that contribute to increased activity of osteoclasts [36, 37]. Decreased growth hormone secretion has also been reported; but primary growth hormone deficiency is encountered only occasionally as basal growth hormone and stimulated growth hormone levels are normal in most HIV-infected children [38, 39].

Secondly, our study noted significant delays in sexual maturation in the HIV-infected subjects using both the pubic hair development and testicular volume. None of the controls were found to be in stage 1 pubic hair development as opposed to fourteen subjects that were in the same stage. This difference statistically implied that the HIV-positive children were about ten times less likely to navigate from stage 1 to stage 2 of pubic hair development compared to their HIV-negative controls. These findings are in keeping with those of studies conducted in the United States [17, 40] and in Uganda and Zimbabwe [18], and underscore the negative effect of the chronicity of HIV infection on the onset, timing, and progression of sexual maturation. In the present study, the HIV-positive children surprisingly showed a tendency to achieve full testicular development (stage 5) earlier than the controls. This observation contrasts with the findings of other studies previously conducted in Europe [41] and the United States [42]. In one of the studies [41], the ages of pubertal stages for HIV-1-infected girls and boys were at the > 97th percentile and 75-97th percentile, respectively of the controls (who were healthy children that provided the reference percentiles). We could not explain this particular disparity between our findings and those of these previous studies based on the available published literature. Non-adjustment for age in our statistical analysis may have contributed to the disparity. Our analysis of covariance showing the effect of two independent variables on testicular volume (after controlling for age) revealed that the covariate had a strong effect on the subjects’ testicular volume. In a Nigerian study conducted more than two decades ago to obtain normative data on the sexual development of Nigerian children, the authors noted that the onset of puberty in boys was between 9 and 15 years of age [43]. Additionally, there was most often a close concordance between stages of testicular and pubic hair development at each age although stages of both features could be entirely discordant, as testicular development was always ahead of pubic hair development. In a more recent Nigerian study conducted four years ago, the concordance between testicular and pubic hair maturation was also reported by other authors, as the mean age of onset of pubic hair (stage 2) was 11 years in boys, while testicular development (stage 2) was seen at a mean age of 11 years [44].

Generally, perinatal HIV-1 infection interferes with sexual maturation although the mechanisms by which this occurs are yet to be fully elucidated. However, the suggested mechanisms as previously mentioned include the dysfunction of the HPG axis [12], and the ‘euthyroid sick syndrome’ [13]. More importantly, it appears that the earlier the onset and severity of HIV infection, the greater the negative effects on pubertal growth: worsened by the vulnerability of adolescents to hormonal changes because of the immaturity of their HPG axis. This may partly explain why the duration of HAART use did not affect the delay in the early stages of pubic hair and testicular volume development in our study subjects.

Our study has some limitations. We used a Prader orchidometer to measure the testicular volume of the study participants. However, an ultrasound-scan measurement of the testicular volume may have provided more reliable data. Secondly, we conducted a cross-sectional study rather than a longitudinal study which would have better described the growth trajectory of perinatally HIV-infected children and their sexual maturation.

Therefore, we recommend a future research direction based on a longitudinal study with multiple interval assessments of sexual maturation throughout adolescence. This prospective study should be explored to better define the age of onset and progression through the different stages of puberty in HIV-infected children.

## Conclusion

Perinatal HIV infection negatively affects physical growth and the onset of pubic hair sexual maturation (PH2) despite the duration of HAART. Although the use of HAART is presumed to limit viral replication and thus improve growth, previous studies show that regimen containing protease inhibitors significantly affected the weight of infected children and did not lead to an overall improvement in their growth [15, 45]. On the other hand, perinatal HIV infection did not influence final testicular maturation (G5) although there was a delay in pubic hair and testicular development for Stages 1, 2, and 3. Thus, we recommend screening for weight deficits and serial assessments of sexual maturation in HIV-infected male children as they approach adolescence. Failure of testicular development by the 14th year of life indicates a delay in puberty. Chronic diseases (such as HIV) and constitutional delay of growth and puberty (a variant of normal physical development) are known causes of this delay. Thus, screening for a delay in puberty in male adolescents with perinatal HIV infection may warrant initial estimation of follicle-stimulating hormone (FSH), luteinizing hormone (LH), and testosterone to exclude a delay related to chronic etiology [46]. If a permanent dysfunction of the HPG axis occurs following HIV infection, the consequential intervention may involve hormone-replacement therapy with testosterone/dihydrotestosterone [47].

## Data Availability

The datasets used and/or analyzed during the current study available from the corresponding author on reasonable request.

## Abbreviations

AIDS: Acquired immune deficiency syndrome.
BAZ: BMI-for-age Z-Score.
BMI: Body mass index.
HAART: Highly active antiretroviral therapy.
HAZ: Height-for-age Z-Score.
HIV: Human immune-deficiency virus.
HPG: Hypothalamic-pituitary-gonadal.
IGFBP-3: insulin-like growth factor-1 binding protein-3.
IGF-1: Insulin-like growth factor-1.
PH: Pubic hair.
PMTCT: Prevention of maternal-to-child transmission.
SPSS: Statistical Package for Social Sciences.
WAZ: Weight-for-age Z-score.
WHO: World Health Organization.
